# Seasonal changes of diagnostic potential in the detection of *Anoplocephala perfoliata* equine infections in the climate of Central Europe

**DOI:** 10.1007/s00436-014-4279-9

**Published:** 2014-12-25

**Authors:** Krzysztof Tomczuk, Krzysztof Kostro, Maciej Grzybek, Klaudiusz Szczepaniak, Maria Studzińska, Marta Demkowska-Kutrzepa, Monika Roczeń-Karczmarz

**Affiliations:** 1 Faculty of Veterinary Medicine, University of Life Sciences in Lublin, 13 Akademicka Street, 20-950 Lublin, Poland; 2 Department of Parasitology and Invasive Diseases, Faculty of Veterinary Medicine, University of Life Sciences in Lublin, 12 Akademicka Street, 20-950 Lublin, Poland

**Keywords:** *Anoplocephala perfoliata*, Equine tapeworms, Seasonal changes, Coproscopic methods, Isophenes

## Abstract

For this study, 724 gastrointestinal tracts of slaughter horses were investigated to determine the prevalence, intensity of *Anoplocephala perfoliata* and tapeworm development stages over the second, third and fourth quarter of 2012 and the first quarter of 2013. For each positive horse, faecal samples were collected from the rectum or small colon for coproscopic examinations. The samples were analysed using dedicated modified sedimentation-flotation methods. In total, 52 horses were infected with *A. perfoliata* in the course of the study, with an overall prevalence of 7.2 %. The prevalence changed over the study period; however, not markedly. The overall mean of *A. perfoliata* abundance was 12.3 (3.23) and did not differ significantly between the quarters. Mean invasion intensity did not differ significantly between the quarters. The quantity of mature tapeworms did not differ significantly over the study period; however, there was a significant difference in the number of immature tapeworms. The highest number of mature tapeworms was found in the first quarter of 2013. The number of detected tapeworm eggs rose significantly over the study period. The total number of tapeworms did not have a significant influence on the presence/absence of detected eggs. However, there was a noticeable difference between the number of mature tapeworms and presence/absence of eggs in faeces. This clearly indicates that the efficacy of the modified sedimentation-flotation method is influenced by seasonality, and therefore the most effective and reliable time for detection of *A. perfoliata* in equines is the first quarter of the year.

## Introduction

Horses are known to be definitive hosts to a variety of internal parasites including both nematodes and cestodes (Uhlinger 1990; Proudman et al. 1998; Love et al. 1999; Gundlach et al. 2004; Andersen et al. 2013; Studzinska et al. 2012). *Anoplocephala perfoliata* invasions represent a major cause of morbidity and mortality in horses thus considerably contributing to economic losses of horse breeders (Nilsson et al. 1995; Kornaś et al. 2007, 2010; Rehbein et al. 2013).

Despite well-developed molecular and serological methods, equine cestodosis is diagnosed via coproscopic methods. This is due to the high cost of reagents, equipment availability, and competence of veterinary surgeons. The sensitivity of sedimentation-flotation methods is comparable to that of serological methods (Traversa et al. 2008), and what is more, the cost of coproscopic methods is much lower than for other methods. Anoplocephalidae are very different from other animal tapeworms species. In *A. perfoliata*, before shearing off from the strobila, gravid proglottids lose eggs via the violable wall of the uterus from the last proglottid (Tomczuk, unpublished observations). Damages of the last gravid proglottid internal structures may reach more than a few proglottids.

Proglottids egested into the environment rarely contain eggs. This causes that eggs are present in animal faeces and expelled with faeces rather than in proglottids. Large volumes of faecal material in horses keep egg concentration in faeces low. This fact has an impact on the efficiency of anoplocephalosis diagnosis. Detection of *A. perfoliata* invasions with the sedimentation-flotation method is possible with minimal intensity of 9–10 tapeworms (Tomczuk et al. 2014), which permits many invasions to go undetected. *A. perfoliata* invasions are responsible for intestinal disorders including ileo-caecal, caeco-caecal, caeco-colic intussusceptions, ileal impactions and spasmodic colics (Beroza et al. 1983; Gawor 1995; Proudman and Holdstock 2000). However, most horses do not show any specific symptoms of invasion, and even with intense tapeworm invasion, no specific clinical symptoms are demonstrated (Veronesi et al. 2009). In race horses, symptoms may include a worse physical state such as exhaustion. This kind of invasion makes breeders less vigilant, who trust the negative result of a parasitological test and therefore choose not to use anthelmintics. *A. perfoliata* invasions may lead to the host’s death by perforating the ceacum without any previous symptoms. This illustrates how dangerous *A. perfoliata* invasions can be.

Anoplocephalosis in equines results from pasture invasions. The seasonal dynamics of *A. perfoliata* invasions may be influenced by external factors such as climatic and environmental conditions. Endoparasite seasonal dynamics in Poland has been described for other parasite species such as gastrointestinal nematodes, lungworms or flukeworms in livestock (i.e. Balicka-Ramisz et al. 2013). Seasonal changes in pasture invasions vary significantly among different geographic locations, especially in locations where the pasture season is periodical. In the active vegetation period, pasture invasions are cyclic, with observable reinvasions and superinvasions. Previous studies (Gundlach et al. 2003, 2004; Tomczuk et al. 2014) reported tapeworm invasions at a different developmental stage, the abundance changing in the course of the year. Taking into consideration the fact that only tapeworms with gravid proglottids produce eggs, the presence of mature tapeworms affect invasion detection. Climate conditions in Central and Eastern Europe, characterized by early and long winters, may have a direct impact on the seasonality of *A. perfoliata* invasions. This is caused by the presence of prepatent and patent periods of invasion at different times of the year. This may also directly affect detection sensitivity of various equine anaplacopheloses. Therefore, the primary aim of this study was to analyse seasonal changes in the sensitivity of the modified sedimentation-flotation method, which is used for detecting *A. perfoliata* invasions, considering the maturity of recovered tapeworms.

## Materials and methods

Parasitological post-mortem examinations of gastrointestinal tracts of 724 slaughter horses were performed to diagnose the intensity of tapeworm invasion over the second, third and fourth quarter of 2012 and the first quarter of 2013. Slaughter horses came from the south-eastern part of Poland. This part of the country is characterized by temperate climate, with points of oceanic climate, but with the favour to continental climate (McKnight and Hess 2000). The range of mean temperatures in January (the coldest month) is from −4 to −5 °C. The range of mean temperatures in July (the hottest month) varies from 18 to 19 °C. The average annual precipitation for the whole country is from 600 to 800 mm. Snow coverage persists for 70–80 days and vegetation period lasts for 210–220 days (Czarnecka 2012).

Species identification and maturity stages of tapeworms were determined considering morphological characteristics and the presence of eggs in the gravid segments (Schuster 1991). For each positive horse (tapeworms present in the caecum), faecal samples were collected from the rectum or small colon for coproscopic examinations. Samples were analysed with modified sedimentation-flotation methods (Gundlach et al. 2003). A 50-g stool sample was homogenized in 400 ml 0.0025 % Tween 80 solution with glass beads in a laboratory shaker for 3 min. The suspension was filtrated using a 200-μm sieve. After removing the solid fraction, the sample was sedimented by centrifugation (3000 rpm for 10 min). After removing the supernatant, the residue was again homogenized in sucrose and NaCl solution with glass beads, and placed in 100-ml tubes for flotation with centrifugation (2000 rpm for 3 min). After 30 min of flotation with a cover slip, the sample was examined under a light microscope for tapeworm eggs. The number of tapeworm eggs was counted from the surface of the cover slip.

### Statistical analysis

Prevalence values (percentage of animals infected) are shown with 95 % confidence limits (95 % CL), the latter having been calculated according to Rohlf and Sokal (1995). All means are reported ± standard error of mean (SEM) unless otherwise stated. All data were tested for normality using Shapiro-Wilk test. The testing of the data for normality revealed no significant departure from normal distribution (Shapiro-Wilk; *P* < 0.0001). Therefore, the data was analysed using nonparametric tests. Prevalence, abundance and intensity of infection and the relationship between the number of mature/immature tapeworms and the number of eggs detected in faeces was analysed using Kruskal-Wallis test. Mann-Whitney *U* test was applied to analyse the impact of the number of the mature/immature tapeworms and the presence/absence of eggs in faeces. Method sensitivity was calculated as described in Tomczuk et al. (2014). All of the statistical analyses were conducted using the R version 2.12.0 and MS Excel, 2010. A probability of <0.05 was considered significant.

## Results

**Fig. 1:**
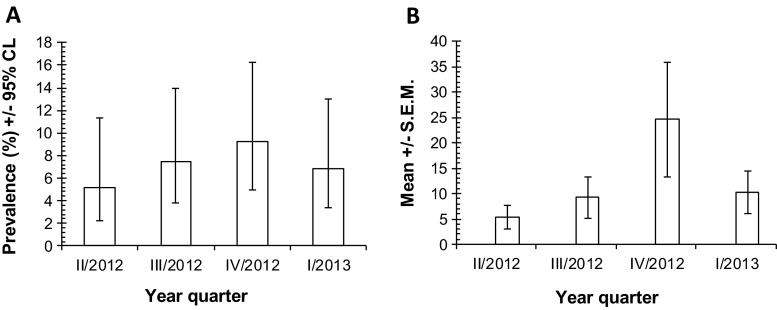
Prevalence (**a**) and abundance (**b**) of *A. perfoliata* over the study period

In total, 52 horses were infected with *A. perfoliata* over the course of the study with an overall prevalence of 7.2 % (5.5–9.3), which changed over the study period, not markedly though. The highest number of infected horses was found in the fourth quarter of 2012, whereas in the other three study periods, the prevalence was comparable (Fig. 1a). The overall mean abundance of *A. perfoliata* was 12.3 (3.23) and did not differ significantly between year quarters. The highest mean worm burden was found in the fourth quarter of 2012 (Fig. 1b). Overall mean tapeworm intensity was 171.9 (39.5). Mean tapeworm intensity did not change significantly over the study period; however, it was the highest in fourth quarter of 2012 (Table 1).

**Table 1:** Results of post-mortem examination of *A. perfoliata* positive horses

Year quarter	No. of horses examined	No. of positive horses	Mean intensity (SEM)^a^	No. of horses infected with mature tapeworms	Number of invasions detected with coproscopic methods	Coproscopic methods sensitivity (%)
II/ 2012	192	10	102.5 (35.3)	5	4	40
III/2012	173	13	122.8 (47.1)	10	7	53.8
IV/2012	183	17	264.7 (110.2)	13	11	64.7
I /2013	176	12	151.4 (48.7)	12	9	75
Total	724	52	171.9 (39.5)	40	31	59.6

^a^Mean intensity was calculated by dividing total number of tapeworms by total number of infected horses

**Fig. 2:**
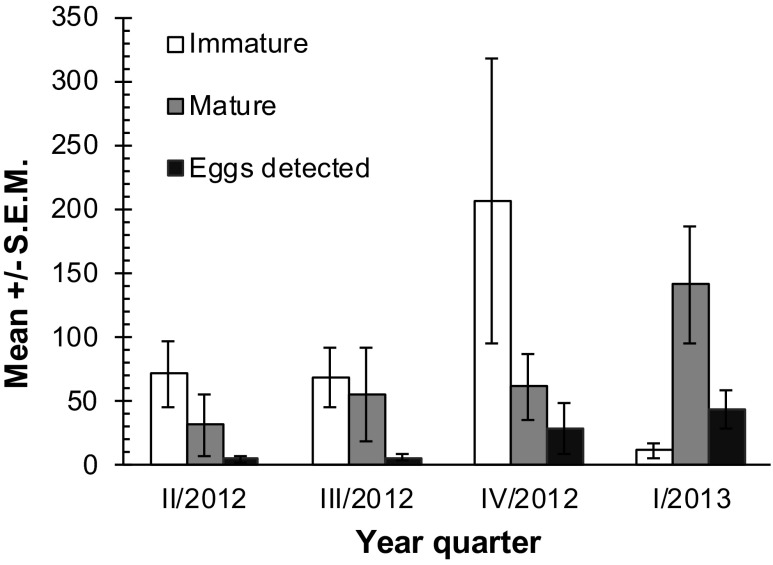
Mean number of tapeworms recovered and number of eggs detected over the study period

In total, 8990 tapeworms were recovered from infected horses (3746 mature, 5244 immature). The number of mature tapeworms did not differ significantly over the study period. However, there was a significant difference in the number of immature tapeworms between year quarters (*χ* ^2^_3_ = 11.56; *P* = 0.009) (Table 1). The maximum number of mature tapeworms was found in the first quarter of 2013, during which horses showed 12-fold higher mature tapeworm burden than for the immature individuals (Fig. 2).

The number of detected tapeworm eggs increased over the study period, with the highest level in the first quarter of 2013. The total number of tapeworms did not significantly influence the presence/absence of detected eggs. However, there was a significant difference between the number of mature tapeworms and presence/absence of eggs in faeces (*U* = 192.5; *P* < 0.0001). The number of detected eggs increased significantly with the number of mature tapeworms (Fig. 2).

## Discussion

The presented results confirm the influence of seasonality of parasitic invasions on the diagnostic potential of *A. perfoliata*. Tests performed on these parasites using the same standards in different parts of the year can vary in efficacy. Seasonality in the course of various parasitic diseases has been the subject of many studies. The seasonality of parasitic invasion is especially noticeable in ectoparasites. The distribution of periods of their increased prevalence is connected with numerous biotic and abiotic factors, and it has undoubtedly an impact on diagnostic potential. Seasonality among endoparasites is prominent in invasions that depend on environmental factors. Many authors have analysed this phenomenon with regard to the most common pasture invasions (gastrointestinal nematodes, *Fasciola hepatica* or lungworms). Their changeable prevalence, invasion intensity and the level of pathogenic influence have been noted. Not in all cases seasonality equally determines detection possibilities. The high proliferation rate of nematodes causes their diagnostic potential to be less dependent on seasonality. This is proved by McMahon et al. (2012), who studied the dynamics of excretion of eggs in gastrointestinal nematodes in ruminants in England, Scotland and Wales, noticing differences in the seasonal dynamics of egg excretion in various types of nematodes, the most visibly for the genus *Nematodirus*. In a similar study, analyses of climatic aspects of gastrointestinal nematode invasions in European sheep allowed Morgan and van Dijk (2012) to point out the considerable role of such physical factors as warmth, cold and moisture on the course of infections. They concluded that unique climatic and environmental conditions affect epidemiological models of certain invasions in particular environments. In the study of susceptibility of ruminants to gastrointestinal nematodes, Fiel et al. (2012) makes a distinction between different periods of the year with regard to the length of incubation of invasive larvae. The time that elapses from the moment when an egg is expelled to its becoming an invasive form L3 varies from 1–2 to 4–6 weeks in different periods of the year. Fiel et al. (2012) also stresses the variable degree of contamination with invasive forms in pastures, the highest in autumn, winter and spring, and the lowest in summer, as the larvae easily die in pastures at this time of the year. The same limiting factor affecting gastrointestinal nematodes in African ruminants living in the wild was demonstrated by Turner and Getz (2010). The seasonality of nematodes is conditional upon the long dry season, which can restrict the development and survival of the invasive forms of the parasites, and this in effect hinders their transmission. The seasonality of nematodes occurs in various climatic zones. Zaffaroni et al. (1999) notices fluctuations of diversity and abundance of abomasal nematodes in the annual cycle in the alpine ibex (*Capra ibex ibex*). In his studies of sheep living in the mild climate of Greece, Theodoropoulos et al. (2000) found the highest contamination of pastures to occur in autumn and winter.

In numerous studies of anoplocephalosis, various authors emphasise the unique, seasonal dynamics of parasitic invasions. It is connected with mites presence in the environment. Oribatid mites (Oribatidae) play a significant role in pedogenesis and as an intermediate hosts in the life cycles of Anoplocephalidae (Shimano 2004). Oribatide mites are present in natural environment with the intensity of few thousands up to hundred of thousands individuals per 1 m^2^. There are more than 500 Oribatide species described in Poland (Niedbała 1980); however, only 20 species have been described as potential vectors of anoplocephalid cestodes (Sengbusch 1977; Haq 1988; Denegri and de Alzuet 1992; McAloon 2004; Shimano 2004). Feeding on organic material, these arthropods eat tapeworms eggs that may develop in their bodies into the invasive form—cysticercoid. Farm animals become infected per os—by eating Oribatide mites with collected food.

Oribatei belong to the group of mites which is numerous and represented by various kinds and species that prefer defined soil types. Number of Oribatide mites in different biocenoses varies due to extrinsic factors such as humidity and temperature. The highest concentrations of mites were described in environments with high humidity and moderate temperatures (Gergócs et al. 2011). These factors may significantly affect prevalence and abundance of anoplocephalid cestodes.

The results of studies carried out only in the European countries indicate that there are three zones having different dynamics of invasion. In the countries of the south, the dry and hot summer season is the limiting factor. Epidemiological studies of equine anoplocephalosis in central Spain demonstrated significant seasonality dependent on humidity in particular seasons (Meana et al. 1998). The highest prevalence occurred in autumn and winter time (37.5 and 32.3 % of infected horses, respectively), while in spring and summer the levels were 9.2 and 10.8 %, respectively. A variable proportion of tapeworms with gravid proglottids was found: from their absence in the summer to 93.5 % presence in springtime. It may be concluded that for Spanish continental and warm climate of central Spain, the highest efficacy of coproscopic tests may be achieved in spring. In countries with a maritime climate (Denmark, England, the Benelux states), seasonal fluctuation is not considerable. The pasture season there lasts throughout the year, and oribatida occur in green plants. This levels out any greater differences in the dynamics of invasions in the year. The cyclical nature of infestations in such regions results in a constant prevalence of mature tapeworms and lack of seasonal fluctuations of egg production. In his autopsy studies of horses in Belgium, Agneessens et al. (1998) demonstrated a prevalence of tapeworm infestations at 28.9 % and found no significant differences in various parts of the year. In Central and Eastern Europe, however, due to low temperatures in winter and warm and dry summer, a specific dynamics of tapeworm infestations is observed. Tomczuk (2012) analysed 1626 alimentary tracts of slaughtered horses, finding a mean prevalence at the level of 6.6 %, with 3.8 % in June and 11.1 % in November. The highest proportion of tapeworms with gravid proglottids was between January and April. Similar results were obtained by Rehbein et al. (2013) in Germany. He confirmed significant seasonality of tapeworm infestations in 400 autopsied horses, with the prevalence of invasion in autumn and winter at 36.1–36.5 %, and only 17.3 and 15.9 % in spring and summer, respectively. Studies conducted in Sweden by Nilsson et al. (1995) demonstrated a high extensiveness of invasion (65 % of horses were infected) if compared to the results gained in other Central European countries (79 tapeworms on average). He also showed a similar seasonality of invasion peaking in the third and fourth quarter. The seasonal dynamics of invasion results in varying diagnostic potential, depending on the amount of tapeworms with gravid proglottids. In his study of the efficacy of coproscopic examinations, Tomczuk et al. (2014) demonstrated that the best method to diagnose anoplocephalosis in horses is to use sedimentation-flotation test, and the detection threshold is a minimum of nine tapeworms with gravid proglottids. A similar sensitivity was confirmed by Williamson et al. (1998), who assumed that a minimum detectable invasion is ten tapeworms in the patent period of invasion. Beelitz and Gothe (1997) examined 100 slaughter horses from the Upper Bavaria region, and reported *A. perfoliata* in 38 horses. Using coproscopic diagnostics, he evaluated the efficacy of the sedimentation-flotation method at 47.4 %. He set the detection threshold at 70 tapeworms irrespective of their maturity. Ihler et al. (1995), in his studies in Norway, found tapeworms in 20 % of autopsied horses, and their invasions were not intensive. The mean intensity of invasions, depending on the region, was 6 and 18 tapeworms. The invasions had a low coproscopic detection rate (11.5 %), so the author did not recommend them for diagnosis. Varying climatic conditions across Europe make parasitic invasions follow different routes depending on environmental factors. Differences are noticeable, hence even within the same country (i.e. Germany), anoplocephalosis will have distinct invasion profiles.

Climatic features have a considerable impact on a region of the world with regard to particular invasions. In temperate zones, the course of a parasitic invasion is determined by low temperatures in winter and inhibited vegetation. In other climatic zones, the level of precipitation and the occurrence the dry season is the critical factor. Climatic conditions cause the invasion potential to vary, producing differences in abundance and clinical course of invasive diseases in diverse parts of the world. This phenomenon determines the occurrence of epidemiological models that are characteristic for particular parasitic diseases in given environments. Looking at the regional, continental or global phenology of parasites, we can compare only those invasions that occur in similar climatic-environmental circumstances, i.e. zones where invasions will have similar conditions of development. Such locations are typically joined into isophenes, which are lines connecting points on the map where the same biological phenomena take place, in this case relating to the life of parasites (Maggenti et al. 2005). In such locations, we can speak of similar threats as well as similar fighting strategies and diagnostic methods related to parasitic invasions. Therefore, comparisons drawn between remote isophenes do not make sense. Seasonality plays a crucial role in the course of many parasitic diseases (Poulin 1998). This applies chiefly to all regions of the temperate zone, where seasons are clearly demarcated. Diagnostically, invasions by *A. perfoliata* are a good example of the above dependencies, as demonstrated by the presented study. Hence, when analysing the results of coproscopic examinations, one must take into consideration the seasonal fluctuations of diagnostic potential, and properly interpret the results, especially negative ones.
